# Cancer Metabolism as a Therapeutic Target and Review of Interventions

**DOI:** 10.3390/nu15194245

**Published:** 2023-10-01

**Authors:** Matthew T. J. Halma, Jack A. Tuszynski, Paul E. Marik

**Affiliations:** 1 Department of Physics and Astronomy, Vrije Universiteit Amsterdam, 1081 HV Amsterdam, The Netherlands; 2 EbMC Squared CIC, Bath BA2 4BL, UK; 3 Department of Physics, University of Alberta, 11335 Saskatchewan Dr NW, Edmonton, AB T6G 2M9, Canada; 4 Department of Data Science and Engineering, The Silesian University of Technology, 44-100 Gliwice, Poland; 5 DIMEAS, Politecnico di Torino, Corso Duca degli Abruzzi 24, I-1029 Turin, Italy; 6 Frontline COVID-19 Critical Care Alliance, Washington, DC 20036, USA

**Keywords:** cancer metabolism, Warburg effect, glycolysis, ketogenic diet, repurposed drugs, lifestyle interventions

## Abstract

Cancer is amenable to low-cost treatments, given that it has a significant metabolic component, which can be affected through diet and lifestyle change at minimal cost. The Warburg hypothesis states that cancer cells have an altered cell metabolism towards anaerobic glycolysis. Given this metabolic reprogramming in cancer cells, it is possible to target cancers metabolically by depriving them of glucose. In addition to dietary and lifestyle modifications which work on tumors metabolically, there are a panoply of nutritional supplements and repurposed drugs associated with cancer prevention and better treatment outcomes. These interventions and their evidentiary basis are covered in the latter half of this review to guide future cancer treatment.

## 1. Introduction

Warburg’s hypothesis for cancer progression is that cancer cells undergo a two-step transformation. First, there is irreversible failure of respiration, and secondly, the cell survives by fermentation energy. Fermentation energy is far less efficient than aerobic glycolysis, producing only 2 ATP for a glucose molecule as opposed to 38 via aerobic glycolysis [1].

Cancerous cells shunt a majority of glucose through the anaerobic pathway [2], and cancerous cells do have enhanced glycolysis [3]. This effect serves as the basis of radiolabeled glucose positron emission tomography (PET) imaging of tumors [4]. Cancerous tumors using anaerobic glycolysis produce waste products and acidify the intracellular space [5,6], which necessitates the evolution of surrounding cells towards acid-resistant phenotypes [7]. Additionally, the hypoxia-resistant phenotype of cancer cells is especially useful for pre-malignant lesions growing further away from blood vessels [7].

To test the validity of the metabolic theory of cancer, nuclear transplantation experiments were performed [8]. These experiments demonstrated that while inserting the nucleus of a cancerous cell into a healthy cell was insufficient to induce the cancer phenotype, inserting the cytoplasm of a cancerous cell, which contains the mitochondria, was sufficient to transform a previously healthy cell into a cancerous cell [8].

Until recently, the Warburg hypothesis had not received much attention for its treatment implications for cancer. Case reports on the ketogenic diet for treatment in cancer were published in the 1990s [9], and a pilot trial of 16 participants was published in 2011 [10]. Adherence to the diet is a common difficulty [10,11]. Given the profusion of interest in ketogenic diets, there are now more keto-friendly foods and cookbooks available, which can help with diet adherence.

Metabolic approaches work systemically [12], as opposed to targeted therapies, which require specific targeting towards an individual’s cancer genetics [13]. Given that the Warburg effect is a hallmark of cancer [14], a reduction in the fuel available to cancer cells can systemically shift the body environment to be more hostile to cancer [15]. 

There are multiple interventions that one can apply, each of which affects a less conducive environment for cancer growth. Therapeutic combinations that stress the cancer through multiple pathways can prevent cancer progression, encouraging reversal and remission. Typically, therapeutic combinations are studied either in isolation or in combination with a few other (often complementary) therapeutic interventions.

Cancer fitness is a multidimensional landscape, and combinations of interventions can be selected to reduce tumor fitness while maintaining the fitness of normal cells. Metabolic reprogramming in cancer cells opens a significant number of therapeutic modalities. Not only does dietary ketosis proportionately disfavor cancer cells from an energy availability standpoint, but it also deprives the cancer cell of the vital building blocks for cell replication [16], and ketones may act independently as anti-oncogenic factors [17,18,19,20]. 

The metabolic shift to glycolysis is also useful for the tumor microenvironment prior to angiogenesis, which is a hypoxic condition [7]. The rapidly dividing nature of cancer cells prioritizes glycolytic metabolism, and the Warburg effect enables faster glucose breakdown [21,22]. Glycolysis provides substrates for nucleic acid biogenesis in rapidly dividing cancer cells [23], and can produce more energy per unit time compared to non-cancerous cells, despite the inefficiencies [24].

Beyond anaerobic glycolysis (i.e., the Warburg effect), other metabolic shifts occur in cancer cells. In a 2016 review, authors Pavlova and Thompson identified six metabolic hallmarks: (1) deregulated uptake of glucose and amino acids, (2) use of opportunistic modes of nutrient acquisition, (3) use of glycolysis/TCA cycle intermediates for biosynthesis and NADPH production, (4) increased demand for nitrogen, (5) alterations in metabolite driven gene regulation, and (6) metabolic interactions with the microenvironment [25]. That is to say that the differences between cancer and healthy cell metabolism are not limited to the Warburg effect. A more recent review adds the following emerging metabolic hallmarks of cancer: an increased need for electron receptors and a greater reliance on oxidative stress protection mechanisms. Additionally, the heterogeneity of metabolic reprogramming, even within a single tumor, is worth considering, as well as the interaction of the tumor with whole body metabolism [26].

The metabolic paradigm of cancer research is still novel and requires much more fervent investigation. However, it demonstrates great therapeutic promise, both in terms of mechanistic understanding and clinical data [27].

Several existing and investigational anticancer agents act on metabolic pathways. Dichloroacetate, for example, inhibits pyruvate dehydrogenase kinase, in turn increasing pyruvate flux into the mitochondria. This promotes glucose oxidation, as opposed to glycolysis [28], which is the primary source of energy for cancer cells [24], thereby decreasing the energy available to cancer cells [29].

Metabolic approaches also demonstrate potential in the adjunctive setting, when combined with other approaches. Combining chemotherapy with a ketogenic diet can enhance the effect of chemotherapy [30,31,32]. Additionally, ketone body metabolism can suppress reactive oxygen species and enhance antioxidant capability [12], which may be a mechanism behind its positive impacts in cancer radiotherapy [33,34]. For the case of immunotherapy, the ketogenic diet may be beneficial in the adjunctive setting [35,36,37].

**Figure 1:**
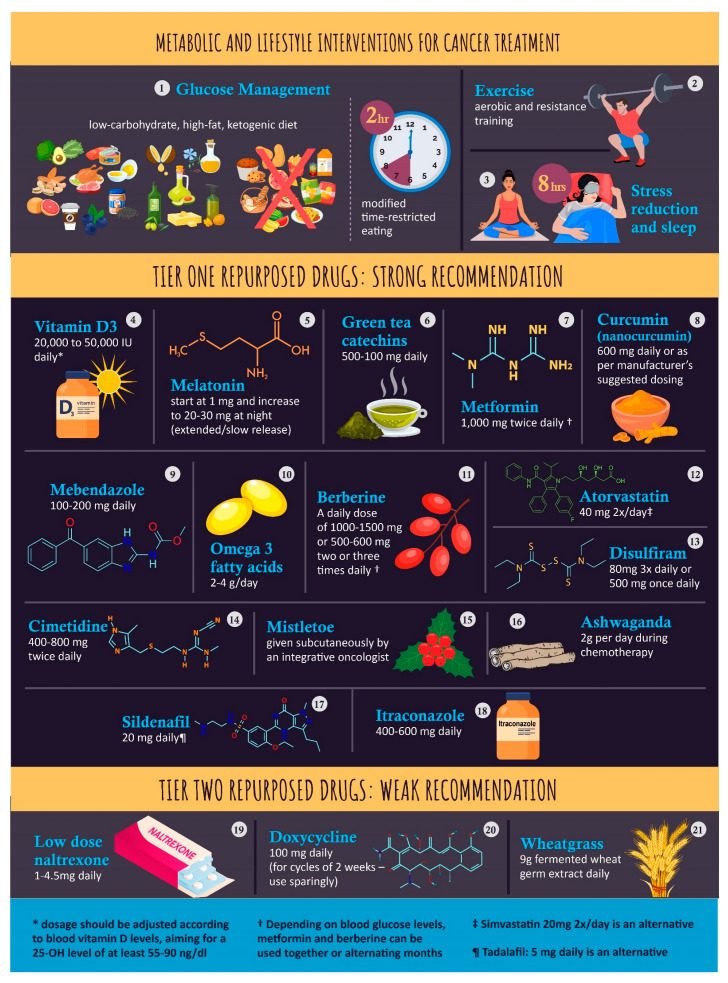
An infographic of Tier 1 (strong recommendation) and Tier 2 (weak recommendation) repurposed drugs.

The interventions covered in this review are summarized in Table 1 and the associated infographic Figure 1. This review is not intended as a guide for treatment, but it may inform cancer treatment using repurposed drugs in the future.

**Figure 2:**
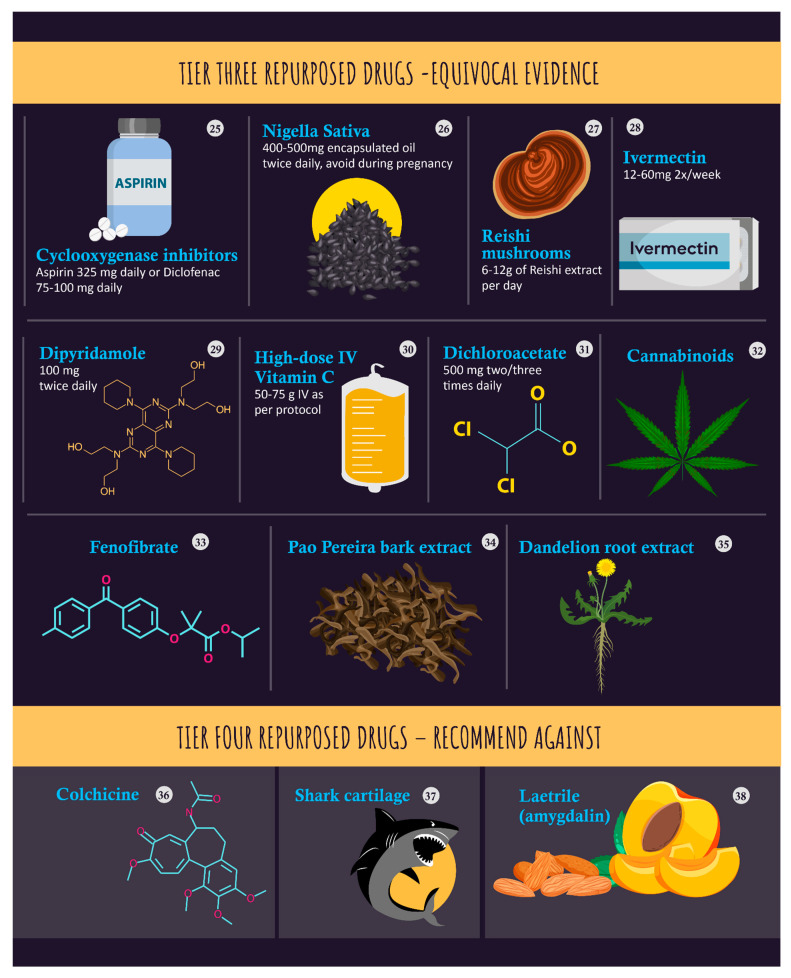
An infographic of Tier 3 (equivocal evidence) and Tier 4 (recommend against) repurposed drugs.

Additionally, non-recommended interventions with a lower evidentiary basis for their efficacy are included in Table 2 and the associated infographic Figure 2.

## 2. Lifestyle Interventions for Preventing and Treating Cancer

### 2.1. Glucose Management and Ketogenic Diet

A carbohydrate-restricted diet, specifically a ketogenic diet, high in saturated fat and Omega-3 fatty acids, is suggested for various health benefits, including its potential role in cancer management [254]. The diet emphasizes avoiding processed foods, particularly those with high glycemic index values, and promotes the consumption of real foods such as vegetables, nuts, fish, chicken, eggs, and certain fruits [254,255,256]. Continuous glucose monitoring is recommended to track blood glucose levels, and a blood ketone meter is advised to confirm the patient’s state of ketosis [257].

To flatten the blood glucose curve, various interventions are recommended, including eating foods in the right order (starting with vegetables, followed by protein and fat, and ending with starches), skipping breakfast, avoiding snacking, and incorporating vinegar or fiber tablets before consuming starchy or sweet foods [258]. Establishing and restoring a normal microbiome is highlighted as an essential aspect of regulating blood glucose levels and improving insulin sensitivity, with suggestions including consuming a diverse range of foods, fermented foods, and prebiotic fiber, and reducing stress and unnecessary antibiotic use [259,260,261,262,263,264,265]. Avoiding seed oils high in linoleic acid is advised, while using healthy oils such as olive oil, avocado oil, coconut oil, flaxseed oil, walnut, and pecan oils, and butter is recommended [266,267,268,269].

Overall, these dietary and lifestyle recommendations aim to support health and potentially impact cancer management positively by optimizing blood glucose levels, promoting a favorable microbiome, and ensuring a balanced intake of fats and oils.

#### 2.1.1. Mechanism

Restricting carbohydrates induces the body to adopt ketosis as its glucose fuel source is restricted and it must necessarily meet energy demands through another mechanism. This mechanism is ketosis, with which it is difficult to meet the metabolic demands of cancers, as these cells have damage to the cellular respiration pathways and ferment glucose preferentially. Ketones also have antitumor effects [17,18,20]. Therefore, being in a state of ketosis is deleterious to the cancer cells while being neutral or even beneficial to the normal cells [12,15]. 

Carbohydrate restriction upregulates ketone production in the liver, either in a state of fasting or a ketogenic diet [270]. Cancer metabolism preferentially uses glucose [271].

The state of ketosis can also positively impact the immune targeting of cancer cells [272]. Reducing angiogenesis is also a promising therapeutic strategy for cancer [273], that is also accomplished in ketogenic diets [274].

#### 2.1.2. Clinical Evidence

Multiple trials have been performed in the last decade showing the clinical benefit of ketogenic diets in the treatment of cancer, showing an antitumor effect in the high majority of trials [275].

#### 2.1.3. Dosing Recommendations

It typically takes two weeks to adjust to the state of ketosis when stating a ketogenic diet [276]. Ketogenic diets (KDs) are very low-carbohydrate, as carbohydrate metabolism will disrupt the onset of ketosis. While standards for the cutoff of carbohydrates vary, keeping the daily intake of carbohydrates below 50 g (not including fiber) is important to maintaining the state of ketosis [41].

Individuals with the following conditions should not embark on the ketogenic diet [277]: primary carnitine deficiency, carnitine palmitoyltransferase deficiency, carnitine translocase deficiency, β-oxidation defects, pyruvate carboxylase deficiency, or porphyria. Other relative contraindications are included in a recent review [278].

It is recommended to start at a higher level of carbohydrates and taper down gradually [279]. Before starting on the KD, it is recommended to perform a consultation which provides relevant information, sets expectations, and provides resources, including cookbooks [278,279,280]. Moreover, 90% of experts recommend multivitamin and mineral supplementation for those using a KD [278]. Specifically, these include calcium and vitamin D3 [278]. Additionally, adequate fluid intake (30–35 mL/kg/d) is recommended, which works out to 2.7 L to 3.2 L per day for a 90 kg person.

These resources are useful, as adherence is often an issue [281], though development on diet formulation helps with adherence, and is similar to a modified Mediterranean diet [282]. A low-carbohydrate diet study managed to maintain 85% adherence over 2 years [283].

For this reason, we recommend the tools of a continuous glucose monitor and ketone test strips, as the latter can demonstrate ketogenic diet adherence by testing β-hydroxybutyrate (BHB) levels [284], which can provide a definite indication that one is in a state of ketosis.

Recommendations for ketogenic diets are provided in the existing literature [277,285,286], and require some tailoring to the individual’s needs. Nutrient deficiencies can manifest over long periods of time on ketogenic diets, including vitamin D, selenium, magnesium, iron, and carnitine [287]. Several practitioners recommend supplementation for nutrients where there is an insufficient level in the diet [288]. Some supplements may also assist in the metabolic shift to ketosis [289], as fatigue is common during the transition [290].

### 2.2. Exercise

Lifestyle modification is crucial for reducing the risk of death from cancer and improving quality of life. This includes exercise, a healthy diet, and stress reduction [49,291]. Patients with cancer and metabolic syndrome face an increased risk of distant metastasis compared to those without the syndrome [292]. Regular exercise, combining aerobic activity and resistance training, is recommended during cancer treatment to improve cardiovascular fitness, cognition, and mood, and reduce fatigue, anxiety, and depression [44,293,294,295,296,297]. Resistance training also helps preserve muscle mass, reducing insulin resistance, improving glucose control, and potentially increasing overall survival, as sarcopenia is a negative prognostic factor in cancer patients [45].

The Combined Aerobic and Resistance Exercise (CARE) Trial demonstrated that a combined dose of 50–60 min of aerobic and resistance exercise performed three times weekly led to better patient-reported outcomes and health-related quality of life compared to performing aerobic exercise alone during breast cancer chemotherapy [298]. Meta-analyses have shown the benefits of exercise in various types of cancer, including breast cancer treated with adjuvant chemotherapy and/or radiotherapy, colorectal cancer treated with chemotherapy, lung cancer treated with chemotherapy, prostate cancer treated with radiation therapy, and hematologic malignancies [293]. Engaging in at least 30 min of moderate-intensity physical activity at least five days a week, or 75 min of more vigorous exercise, along with two to three weekly strength training sessions, is encouraged for patients [44,49]. There is evidence of an inverse dose–response effect between hours per week engaged in physical activity and breast cancer mortality, indicating that more hours of exercise have increased benefits [47,48]. Walking, particularly in the sunshine, is beneficial for physical, emotional, and psychological well-being [299,300].

### 2.3. Stress Reduction and Sleep

Psychosocial stress is associated with a higher incidence of cancer and poorer survival in cancer patients [51]. To reduce stress, patients are advised to engage in stress-reducing activities like meditation, yoga, and mindfulness exercises, along with getting at least 8 h of high-quality sleep [51,301,302,303,304,305,306]. Ashwagandha, an adaptogenic herb, has been proven to be safe and effective in combating stress and improving sleep quality [307,308,309]. In randomized controlled trials, Ashwagandha extract significantly reduced stress levels and cortisol levels, and improved cognition and mood [310]. Meta-analyses also demonstrated that Ashwagandha supplementation significantly reduced anxiety and stress levels compared to placebo, with an optimal dosage of up to 12,000 mg daily for anxiety and 300–600 mg daily for stress [144,311]. However, caution should be exercised as Ashwagandha can activate the immune system and should not be used with immunosuppressive drugs or during pregnancy and breastfeeding [310].

Adequate and high-quality sleep is crucial for neural development, learning, memory, and cardiovascular and metabolic regulation [55]. Disruptions in sleep are associated with a greater cancer risk [312]. Additionally, in those receiving treatment for cancer, sleep disruptions are common [54,313]. For healthy individuals, the National Sleep Foundation recommends seven to nine hours of sleep for younger adults and seven to eight hours for older adults [56]. Healthy sleep is characterized by good quality, indicated by factors such as short sleep latency, minimal awakenings during the night, and high sleep efficiency [314]. Insomnia, defined as difficulty initiating or maintaining sleep, is associated with daytime symptoms like fatigue, cognitive impairment, or depression [315]. Short sleep duration, less than six hours per day, is associated with increased mortality [316]. Ashwagandha supplementation has been found to improve sleep, particularly in adults with insomnia, with positive effects on sleep quality, sleep onset latency, total sleep time, wake time after sleep onset, and sleep efficiency [315]. The optimal treatment dosage is >600 mg daily for more than 8 weeks [315].

## 3. Recommended Supplements and Medications for the Treatment of Cancer

This review summarizes the most promising repurposed drugs for treating cancer, including their mechanism of action, clinical efficacy (if available), and dosing considerations, including safety. These are summarized in Table 1.

For evidence, we include first meta-analyses of clinical trials. If these are not available, we then include individual clinical trials. If these are not available, we include case series, and then case studies. If human studies are not available, we rely on preclinical evidence. 

The prices of the different compounds have been compiled in Supplementary Table S1, which includes their prices through common bulk suppliers for natural products, supplements, and nutraceuticals. For the case of drug prices, US prices are found using the website https://www.pharmacychecker.com/ (accessed on 11 September 2023).

### 3.1. Vitamin D

Vitamin D is synthesized in the human skin through the influence of UV B radiation and is then converted into the active form, 1,25-dihydroxyvitamin D3 (calcitriol), in the kidney [317,318,319]. 25-hydroxyvitamin D3 (25(OH)D3) is considered the best indicator of vitamin D status, with a level > 30 ng/mL considered normal, 20–30 ng/mL considered insufficient, and <20 ng/mL considered deficient [318,319,320]. Recent data suggest that a level > 50 ng/mL is desirable, and, ideally, targeting a level between 55 and 90 ng/mL is preferable [317,321,322,323]. Adequate vitamin D supplementation is important to achieve optimal levels in patients with low vitamin D levels, and using 50,000 IU D3 capsules in divided doses over a few days is recommended [317,322,323] (Supplementary Table S1).

Vitamin D plays a critical role in various physiological pathways, including energy metabolism, immunity, and cellular growth [324]. It has pleiotropic functions and regulates over 1200 genes within the human genome, with a significant role in the modulation of the immune system [317,325,326]. Observational and randomized controlled studies indicate that a low vitamin D status is associated with higher mortality from conditions like cancer and cardiovascular disease [327,328]. Vitamin D deficiency increases the risk of breast, colon, prostate, and other cancers, while supplemental vitamin D intake has an inverse relationship with cancer risk [318,329]. Higher latitudes are associated with increased risk of vitamin D deficiency and various cancers, with vitamin D supplementation likely playing a crucial role in cancer prevention [318,330]. Achieving a vitamin D level of 80 ng/mL may reduce cancer incidence rates by 70% [331].

#### 3.1.1. Mechanism

Vitamin D exhibits diverse antineoplastic activity through various pathways. It binds to the vitamin D receptor and induces differentiation and apoptosis; it also inhibits cancer stem cells, proliferation, angiogenesis, and metastatic potential [332]. It promotes apoptosis of cancer cells, inhibits WNT-β catenin signaling, and has anti-inflammatory effects by downregulating nuclear factor-Κβ and inhibiting cyclooxygenase expression [333,334,335]. Vitamin D also regulates cell cycle, growth factor pathways, and immune responses against tumor cells [331,336]. It triggers autophagic death, promotes cell differentiation, and exhibits anti-angiogenic effects [331,337,338]. These mechanisms collectively contribute to vitamin D’s anticancer effects and may help reduce cancer incidence and mortality [330].

#### 3.1.2. Clinical Evidence

Multiple studies indicate that a significant proportion of cancer patients are vitamin D-deficient (level < 20 ng/mL) and that higher plasma 25-hydroxyvitamin D levels are associated with improved survival in colorectal, breast, gastric, and lymphoma cancer patients [328,332,339,340,341,342,343,344,345]. Meta-analyses and clinical trials demonstrate that vitamin D supplementation may reduce cancer mortality and improve survival in cancer patients, especially when used in combination with chemotherapy [346,347,348,349,350,351,352]. SUNSHINE, a clinical trial on metastatic colorectal cancer patients, showed that “high-dose” vitamin D3 (aiming for a level of >50 ng/mL) combined with standard chemotherapy resulted in improved progression-free survival compared to standard-dose vitamin D3 [332]. Adequate vitamin D dosing aiming for a level between 55 and 90 ng/mL may have additional benefits in cancer patients [332]. Vitamin D supplementation is suggested to have additive or synergistic effects when combined with conventional chemotherapy [353,354].

#### 3.1.3. Dosing Recommendation

Vitamin D supplementation is likely beneficial for most types of cancers, especially breast, colorectal, gastric, esophageal, lung, and prostate cancer, lymphomas, and melanoma. Due to the severe vitamin D deficiency observed in most cancer patients, a high loading dose of vitamin D is recommended, followed by dose titration aiming for a level of >50 ng/mL (target 55–90 ng/mL). Some types of cancer may require higher levels, up to 150 ng/mL, to halt growth and metastasis. Daily doses of 20,000 to 50,000 IU/day are suggested until the desired vitamin D level is reached. It is important to monitor vitamin D levels to ensure appropriate maintenance dosing, and daily dosing of vitamin D3 is preferred over large intermittent bolus dosing. Vitamin K2 (menaquinone (MK4/MK7)) and magnesium are recommended in conjunction with high vitamin D doses (>8000 IU/day). Measuring parathyroid levels and calcium levels can help titrate the vitamin D dose according to the Coimbra Protocol [64,65].

### 3.2. Melatonin

Melatonin, a lipophilic molecule synthesized by the pineal gland with a circadian pattern, exhibits elevated levels at night and contributes to homeostatic metabolic rhythms and disease protection [71]. It acts through MT1 and MT2 receptors found throughout the body, functioning as a potent antioxidant and playing a crucial role in normal mitochondrial function and oxidative phosphorylation [355]. Exposure to light at night can disrupt melatonin production and the circadian rhythm, and melatonin levels decrease with age after 40 [356]. Melatonin’s widespread biological effects are facilitated by its receptors, and it is also produced in mitochondria under near-infrared irradiation, further adding to its diverse properties [357,358].

#### 3.2.1. Mechanism

Low melatonin levels have been associated with cancer development, and various studies have shown reduced melatonin levels in cancer patients compared to healthy individuals of the same age [356]. Disruption of nocturnal melatonin secretion, as seen in night shift workers, is linked to a modestly increased risk of breast and other cancers [359,360], and a long-term study found elevated breast cancer incidence among female airline cabin crew. Experimental models have demonstrated melatonin’s broad spectrum of anticancer activity, involving cytotoxic, anti-mitotic, and pro-apoptotic actions in breast cancer cells, primarily mediated by the MT1 membrane receptor [66,67]. Melatonin inhibits cancer stem cell proliferation, reduces Ki67 and matrix metalloproteinase 9 expression [361], and switches cancer cells from anaerobic glycolysis to oxidative phosphorylation, slowing their proliferation, reducing metastatic potential, and inducing apoptosis [362,363]. Additionally, melatonin exhibits anti-angiogenic effects, modulates the PI3K and MAPK signaling pathways [361], and stimulates T cell and natural killer cell production while reducing regulatory T cells [364,365].

#### 3.2.2. Clinical Evidence

In addition to case studies [366,367], the clinical benefit of melatonin in patients with cancer is supported by the highest level of evidence, namely, meta-analyses of RCTs [368,369]. Seely et al. systematically reviewed the effects of melatonin in conjunction with chemotherapy, radiotherapy, supportive care, and palliative care on 1-year survival, complete response, partial response, stable disease, and chemotherapy-associated toxicities [369]. This analysis included 21 randomized studies all of which studied solid tumors. The pooled relative risk (RR) for 1-year mortality was 0.63 (95% CI = 0.53–0.74; *p* < 0.001). Improved effects were found for complete response, partial response, and stable disease. In trials combining melatonin with chemotherapy, adjuvant melatonin decreased 1-year mortality (RR = 0.60; 95% CI = 0.54–0.67).

#### 3.2.3. Dosing

Melatonin may be active in several cancers including cancers of the breast, ovary, pancreas, liver, kidney, mouth, stomach, colon/rectum, brain, lung, prostate, head and neck, and various leukemias and sarcomas [66,67]. Providers should advise patients to begin with 1 mg at night; a slow-release/extended-release preparation is suggested to minimize REM sleep-induced nightmares (best taken an hour before retiring). The dose should be increased up to 20–30 mg, as tolerated [71].

### 3.3. Green Tea

#### 3.3.1. Mechanism

The biochemical impact of green tea on cancer is multifarious, as green tea is a combination of many bioactive compounds. Of particular interest are catechins, of which epigallocatechin gallate (EGCG) is the most abundant [370]. Green tea catechins (GTCs), especially EGCG, have demonstrated anticancer effects in various experimental models by inhibiting cancer growth and modulating multiple signal pathways involved in cancer cells [371,372,373]. EGCG inhibits mitochondrial glutamate dehydrogenase (GDH) [374] and interferes with the VEGF, STAT3, MAPK, and Wnt pathways, leading to the suppression of angiogenesis and tumor cell proliferation [375]. It also suppresses invasion and metastasis by inhibiting MMP activities and promotes the tissue inhibitor of MMP expression [375]. Moreover, GTCs alter the tumor microenvironment, enhancing anticancer immunity by increasing active cytotoxic T lymphocytes and switching “cold” tumors to “hot”, with improved anti-tumor immune therapeutics [376]. EGCG also downregulates the TLR-4 signaling pathway implicated in cancer cachexia [377]. Green tea extract has been shown to suppress cancer stem cells [73,74]. GTCs may have synergistic anticancer activity when combined with other phytochemicals, such as resveratrol [378].

#### 3.3.2. Clinical Evidence

Numerous experimental models and epidemiological data support the anticancer effects of green tea catechins (GTCs). A meta-analysis showed an inverse association between tea catechin intake and various cancers [370], and another meta-analysis demonstrated that GTCs reduced the risk of gastrointestinal, breast, gynecological, leukemia, lung, and thyroid cancers [379]. Case reports and clinical studies also observed positive outcomes in patients with B cell malignancies and chronic lymphocytic leukemia (CLL) treated with GTCs [380,381]. In a randomized trial, GTCs significantly reduced the risk of prostate cancer [382,383]. Green tea catechins may be effective against various tumors, including prostate, breast, uterus, ovary, colorectal, glioma, liver, gallbladder, melanoma, and lung cancers [370].

#### 3.3.3. Dosing 

Green tea catechins are recommended to be taken at a dose of 500–1000 mg/day during or after a meal to minimize the risk of liver toxicity [78]. The US Pharmacopeia Dietary Supplement Information Expert Committee has concluded that green tea extract is safe when used and formulated appropriately, but regular liver function tests are advised for those taking it, and caution should be exercised in patients with underlying liver disease [384].

### 3.4. Metformin

#### 3.4.1. Mechanism

Metformin exhibits anticancer activity through direct effects on cancer cells [385], including inhibition of the AMPK/mTOR pathway [80], as well as indirect effects on the host through blood glucose-lowering properties and anti-inflammatory effects. It inhibits complex I of the electron transport chain, forcing cancer cells to rely on glycolysis for ATP synthesis [79]. Metformin activates AMPK, leading to the suppression of protein synthesis and cell development, ultimately reducing mTOR action [386]. Additionally, metformin upregulates PGC-1, which is involved in mitochondrial biogenesis, and interacts with the SIRT1 pathway, connecting metabolism with cell proliferation [387]. Moreover, metformin regulates the EGFR and IGFR pathways, which play vital roles in cell growth, proliferation, and metabolic processes, suggesting its potential to exert an antitumor effect [387]. Furthermore, metformin suppresses cancer stem cells, offering a unique approach in targeting the root of cancer [388].

#### 3.4.2. Clinical Evidence

Meta-analyses and observational studies have shown that metformin plays a significant role in the primary prevention of cancer, reducing overall cancer incidence [389,390]. It has been associated with improved survival and reduced mortality in patients with various cancers, including colorectal, lung, breast, and prostate cancer [391,392,393]. Moreover, metformin demonstrated significant benefits as an adjunctive treatment for colorectal and prostate cancer, especially in those undergoing radical radiotherapy [394].

#### 3.4.3. Dosing

Metformin shows a broad spectrum of anticancer activity and may be beneficial in preventing various malignancies, including breast, pancreatic, gastric, colorectal, endometrial, prostate, non-small-cell lung cancer (NSCLC), and bladder cancers [387,392,394,395,396,397,398,399]. The recommended dose is 1000 mg twice daily, and it is considered a safe drug with few side effects [400]. However, prolonged use may lead to vitamin B12 deficiency [401], so supplementation is suggested [402]. Caution should be exercised when combining metformin with berberine, as it may cause very low blood glucose levels [403]. Close monitoring is advised, and alternating metformin and berberine monthly may be considered if low glucose occurs. 

### 3.5. Curcumin

Curcumin, popularly called “curry powder” or turmeric, is a polyphenol extracted from Curcuma longa. Curcumin has antioxidant, anti-inflammatory, antimicrobial, antiviral, and anticancer properties [87].

#### 3.5.1. Mechanism

Curcumin, a bioactive compound found in turmeric, exhibits a wide range of anticancer effects by targeting multiple cell signaling pathways in cancer cells [87]. It interferes with the cell cycle, apoptosis, proliferation, survival, invasion, angiogenesis, metastasis, and inflammation [404]. Curcumin suppresses the activity of NF-κB, a key regulator of cancer-related processes, and inhibits STAT3 activation, which promotes cancer growth and survival [405,406]. It downregulates HER2-tyrosine kinase and interferes with EGFR signaling, inhibiting breast cancer cell growth and proliferation [407,408,409]. Curcumin induces apoptosis and inhibits angiogenesis, even in the hypoxic tumor microenvironment, and also shows activity against cancer stem cells [410]. It triggers apoptosis through ROS-mediated ER stress and mitochondrion-dependent pathways and acts on the Wnt/-catenin pathway [411]. Overall, curcumin demonstrates a promising potential as a natural anticancer agent with multiple mechanisms of action [412].

#### 3.5.2. Clinical Evidence

The clinical use of curcumin for its broad anticancer activities has been limited by its poor bioavailability, attributed to low absorption, extensive biotransformation, and rapid elimination [404]. Various curcumin analogs and drug delivery systems are being investigated to enhance bioavailability [404]. Despite limited clinical studies, some have shown promising results. In patients with multiple myeloma, the addition of curcumin to standard treatment increased remission rates and reduced inflammatory markers [413]. In metastatic colorectal cancer, curcumin as an adjunctive therapy to chemotherapy improved overall survival. In advanced pancreatic cancer, a phytosome complex of curcumin showed a response rate and disease control rate [414,415]. In advanced metastatic breast cancer, intravenous curcumin in combination with paclitaxel resulted in a significantly higher objective response rate [416]. Dose escalation studies demonstrated that daily doses of up to 10 g of curcumin were well tolerated in patients with breast and prostate cancer. 

#### 3.5.3. Dosing

Curcumin (turmeric) may be beneficial for various types of cancers, including colorectal, lung, pancreatic, breast, prostate, chronic myeloid leukemia, liver, gastric, brain tumors, ovarian, skin, head and neck, lymphoma, esophageal cancer, and myeloma [87,415,416]. Its clinical use has been limited by poor solubility, absorption, and bioavailability [412,417]. Formulating curcumin into nanocarrier formulations can overcome these limitations and enhance therapeutic efficacy [418]. It is recommended to use USP-grade supplements to ensure product quality. A common therapeutic dose is 400–600 mg per day [90]. Curcumin is generally safe with doses of up to 8–10 g/day, but diarrhea may occur if the daily dose exceeds 4 g [419]. Long-term use should be monitored for potential hepatotoxicity, and curcumin may interact with certain drugs, including anticoagulants and antibiotics [417,420]. 

### 3.6. Mebendazole

#### 3.6.1. Mechanism

Mebendazole (MBZ), originally developed to treat parasitic worms, disrupts cellular microtubule formation in abnormal cancer cells, inhibiting tumor progression factors such as tubulin polymerization, angiogenesis, pro-survival pathways, matrix metalloproteinases, and drug resistance proteins [421,422]. It targets cancer stem cells, inhibits the Hedgehog pathway, activates apoptosis through Bcl-2 inactivation and caspase activation, and modulates the MAPK pathway [423,424]. MBZ interferes with cancer cells’ glycolysis-dependent metabolism and inhibits mitochondrial oxidative phosphorylation. It crosses the blood–brain barrier, slowing the growth of gliomas, and enhancing sensitivity to chemotherapy and radiotherapy [425]. MBZ can also sensitize cancer cells to conventional therapy, making it a potential adjuvant therapeutic in combination with traditional chemotherapy. When combined with low-dose chemotherapy, MBZ may also help destroy tumor-associated macrophage cells, creating an unfavorable environment for cancer growth [426].

#### 3.6.2. Clinical Evidence

Clinical studies on the use of benzimidazoles in cancer are limited to a few case reports [92,93] and a small case series [94]. Mebendazole is part of the multidrug cocktail used in the METRICS study [427]. The use of benzimidazoles, especially fenbendazole, has gained attention as a repurposed drug for cancer, following the reported experience of Joe Tippens, who achieved apparent remission from non-small-cell lung cancer with extensive metastatic disease after taking fenbendazole and nanocurcumin [428]. However, further research is needed to confirm the efficacy and safety of these treatments.

#### 3.6.3. Dosing

Mebendazole has demonstrated potential benefits in a wide range of cancers, including NSCLC, adrenocortical, colorectal, chemo-resistant melanoma, glioblastoma multiforme, colon, leukemia, osteosarcoma/soft tissue sarcoma, acute myeloid sarcoma, breast (ER+ invasive ductal), kidney, and ovarian carcinoma [96,421,422,426]. A suggested dose of Mebendazole is 100–200 mg/day, and it can be obtained at a more affordable cost from international compounding pharmacies in India (27 c for a 100 mg tablet) [96].

### 3.7. Omega-3

Polyunsaturated fatty acids (PUFA), including alpha-linolenic acid (ALA), eicosapentaenoic acid (EPA), and docosahexaenoic acid (DHA), have been extensively studied for their therapeutic effects against various human diseases, including cardiovascular and neurodegenerative diseases, and cancer [96]. These studies have shown the clinical usefulness and safety of these natural substances. Recent research has also demonstrated the potential of omega-3 FAs in improving outcomes in certain types of cancer, enhancing the efficacy and tolerability of chemotherapy, and improving quality of life indicators. Additionally, omega-3 FAs have been found to have a positive impact on cancer cachexia [96].

#### 3.7.1. Mechanism

Omega-3 fatty acids (omega-3 FAs) have been proposed to exhibit four main antineoplastic activities: modulation of cyclooxygenase (COX) activity, alteration of membrane dynamics and cell surface receptor function, increased cellular oxidative stress, and the production of novel anti-inflammatory lipid mediators such as resolvins, protectins, and maresins [97,429]. Omega-3 FAs compete with omega-6 fatty acids (omega-6 FAs), particularly linoleic acid (LA), which is associated with a pro-inflammatory response. The balance between omega-3 and omega-6 FAs in the diet influences cancer progression, with omega-3 FAs promoting tumor cell self-destruction and limiting cancer expansion, while LA supports tumor cell survival. Omega-3 FAs affect cancer cell replication, cell cycle, and cell death, and have been shown to sensitize tumor cells to anticancer drugs [430]. They also modulate various signaling pathways, including NF-κB, Notch, Hedgehog, Wnt, and mitogen-activated protein kinases (MAPKs), and suppress the formation of pro-inflammatory prostaglandins, thereby influencing inflammatory response, cell growth, apoptosis, angiogenesis, and metastasis [431,432]. Omega-3 FAs have been found to induce apoptosis in breast cancer cells, block the activity of colon cancer stem cells, and exhibit potential anticancer effects [433].

#### 3.7.2. Clinical Evidence

Clinical studies have shown promising results regarding the beneficial effects of omega-3 fatty acids (omega-3 FAs) in reducing the risk of developing cancer and improving outcomes in cancer patients. Prospective randomized controlled trials (RCTs) and cohort studies have demonstrated that the intake of omega-3 FAs is associated with a reduced risk of breast cancer, colorectal neoplasia, and prostate cancer-related death [102,434,435,436]. Additionally, supplementation with omega-3 FAs, particularly eicosapentaenoic acid (EPA) and docosahexaenoic acid (DHA), has been shown to enhance the efficacy of chemotherapy in breast cancer and non-small-cell lung cancer patients [437], improve survival in leukemia and lymphoma patients, and ameliorate cancer cachexia symptoms, leading to an improvement in quality of life and duration of survival [438,439,440]. These findings suggest that omega-3 FAs may serve as a potential complementary or adjuvant therapy in cancer management.

#### 3.7.3. Dosing

Omega-3 fatty acids may be beneficial in breast cancer, colorectal cancer, leukemia, gastric cancer, pancreatic cancer, esophageal cancer, prostate cancer, lung cancer, and head and neck cancer when taken at a dose of 2–4 g daily, but caution should be exercised in patients on anticoagulants due to the potential risk of bleeding [102].

### 3.8. Berberine

#### 3.8.1. Mechanisms

Berberine exhibits multiple anticancer mechanisms, including reducing cancer cell growth, preventing metastasis, and inducing apoptosis [441]. It may also enhance the effects of other cancer treatments, by sensitizing cells to chemotherapeutic drugs via interactions with DNA repair proteins [441]. It achieves these effects through various pathways, such as upregulating miR-214-3p and downregulating SCT protein levels, inhibiting telomerase activity, deactivating MAPK signaling, and modulating the AMPK-p53, PI3K/AKT/mTOR, and miR19a/TF/MAPK pathways [442,443,444]. Additionally, berberine influences the gut microbiota by increasing the Firmicutes/Bacteroidetes ratio and the relative abundance of specific bacteria. These actions contribute to its antibacterial properties, which further impact the tumor microenvironment [445]. Berberine’s ability to enhance radiation sensitivity and the effects of anticancer drugs like cisplatin, 5-fluorouracil, doxorubicin, niraparib, and icotinib highlights its potential as an effective adjunct therapy for cancer treatment [446].

#### 3.8.2. Clinical Evidence

While there are limited clinical data on the benefits of berberine, a randomized, double-blind study demonstrated that berberine in a dose of 300 mg twice daily significantly reduced the risk of recurrent colorectal adenomas following polypectomy [447].

#### 3.8.3. Dosing

Berberine demonstrates anticancer effects in various cancer types, including breast, lung, gastric, liver, colorectal, ovarian, cervical, and prostate [441]. A suggested daily dose of 1000–1500 mg is recommended, taken in divided doses throughout the day. Caution should be exercised when using berberine with certain medications [448], and its use should be avoided in combination with cyclosporine [449]. Regular monitoring of blood glucose levels is important, especially when combined with other diabetes medications like metformin [403]. Patients scheduled for surgery should inform their anesthesia team about berberine use, as it may need to be discontinued one week before the procedure [442].

### 3.9. Atorvastatin

#### 3.9.1. Mechanism

Statins exert direct anticancer effects by inhibiting the cholesterol-producing enzyme HMG CoA, leading to reduced availability of cholesterol needed for cell membrane formation in rapidly proliferating tumors [450,451]. This limitation of cellular proliferation may hinder cancer growth and metastasis. Statins also modulate gene expression, promote cancer cell death through caspase reactivation and upregulation of PPARγ, decrease cell surface glucose receptors, and deplete isoprenoids critical for controlling cancer cell growth and spread [452,453].

#### 3.9.2. Clinical Evidence

Clinical studies have consistently shown that lipophilic statins, such as simvastatin, reduce the incidence and mortality of various cancers, including prostate, breast, colorectal, hepatocellular, and lung [111,116]. Statin use has been associated with improved recurrence-free survival and reduced cancer-specific mortality in different cancer types [113,118].

#### 3.9.3. Dosing

Studies have used 40 mg 2x/day as a dosage for atorvastatin [326]. An alternative is 20 mg 2x/day as dosage for simvastatin [330].

### 3.10. Disulfiram

Disulfiram (DSF) inhibits aldehyde dehydrogenase, leading to acetaldehyde accumulation and unpleasant effects when alcohol is consumed, making it an anti-alcoholism drug; however, it has been repurposed as a potent cancer treatment, showing anti-tumor effects in preclinical studies and recent success in treating seven types of cancer in humans [454].

#### 3.10.1. Mechanism

Disulfiram (DSF) exhibits multiple anticancer pathways, including inhibition of NF-kB signaling, proteasome activity, and ALDH, induction of ER stress and autophagy, and targeting of cancer stem cells [454]. DSF’s cytotoxicity relies on copper (Cu), as DSF/Cu induces ROS production and inhibits NF-κB, activating pro-apoptotic pathways while downregulating anti-apoptotic pathways [124,455]. DSF also forms a complex with Cu, leading to DNA repair pathway downregulation [456]. Clinical trials have shown that DSF/Cu exerts antitumor effects in various cancers, such as head and neck squamous cell carcinoma, glioblastoma, and others, effectively inducing apoptosis in cancer cells and synergistically enhancing the efficacy of conventional chemotherapeutic drugs when administered in combination [457,458].

#### 3.10.2. Clinical Evidence

In a double-blind trial with breast cancer patients, treatment with sodium ditiocarb (diethyldithiocarbamate) significantly improved overall survival (81% vs. 55%) and disease-free survival (76% vs. 55%) compared to the placebo group [459]. A phase IIb clinical trial showed that adding DSF to cisplatin and vinorelbine combination regimen prolonged survival in newly diagnosed non-small-cell lung cancer patients, and DSF plus copper added to temozolomide appeared to prolong disease-free survival in glioblastoma patients [128,460].

#### 3.10.3. Dosing

DSF may be beneficial in treating breast, lung, pancreatic, prostate, liver, and ovarian cancer, as well as acute myeloid leukemia, glioblastoma, and melanoma, with a particular role in glioblastoma patients [124,454]. The recommended dosing for DSF is generally 80 mg three times a day or 500 mg once daily, and copper should be added at a dose of 2 mg three times a day [128,129].

### 3.11. Cimetidine

#### 3.11.1. Mechanism

Cimetidine, commonly used to treat ulcers and gastroesophageal reflux disease, exhibits multiple anti-tumor effects [421], including anti-proliferative actions by blocking H2 receptors and inducing apoptosis, immunomodulation by decreasing immunosuppressive cells and increasing natural killer cell activity, anti-cell adhesion effects, and anti-angiogenic effects through the downregulation of angiogenesis-promoting factors [461,462].

#### 3.11.2. Clinical Studies

The clinical benefits of cimetidine in cancer patients are not extensively studied, with most research focusing on post-operative colorectal surgery patients [421]. However, a Cochrane meta-analysis of five studies involving 421 patients prescribed cimetidine as an adjunct to curative surgical resection of colorectal cancers showed a significant improvement in overall survival (HR 0.53; 95% CI 0.32 to 0.87) [132]. In addition, two small series of patients with melanoma treated with a combination of cimetidine and interferon demonstrated positive clinical responses, including complete regression, partial regression, and prolonged disease stabilization [463]. Furthermore, a report from Denmark found that oral cimetidine, given at a dose of 400 mg twice daily for 2 years, was associated with increased median survival in gastric cancer patients compared to the placebo group (450 days vs. 316 days, *p* = 0.02), and higher relative survival rates were observed in the cimetidine-treated patients at 1 year (45% vs. 28%) [131].

#### 3.11.3. Dosing

Cimetidine may be beneficial in patients with colorectal cancer, melanoma, gastric cancer, pancreatic cancer, ovarian carcinoma, prostate cancer, Kaposi’s Sarcoma, salivary gland tumors, renal cell carcinoma, breast cancer, glioblastoma, and bladder cancer [421]. The standard dosing of cimetidine is 400 mg twice daily, and it is generally well-tolerated, with the most common side effect being gynecomastia [134].

### 3.12. Mistletoe

The European white-berry mistletoe (*Viscum album* L.) is commonly used in continental Europe as an adjunctive treatment for cancer patients, with mistletoe extracts administered subcutaneously or intravenously to reduce disease- and treatment-related symptoms and improve quality of life [464].

#### 3.12.1. Mechanism

Mistletoe extracts exhibit various anticancer effects, including antitumor, apoptotic, anti-proliferative, and immunomodulatory activities. These effects are attributed to the presence of biologically active molecules such as lectins, flavonoids, viscotoxins, and polysaccharides, which mediate immunological activities, increase natural killer cytotoxicity, induce apoptosis, and interfere with protein synthesis in cancer cells [135,465,466,467]. Additionally, mistletoe has been found to enhance chemosensitivity in both cisplatin-sensitive and resistant ovarian cancer cells and may possess anti-angiogenic properties [136,468].

#### 3.12.2. Clinical Evidence

Over 50 prospective studies, including more than 30 randomized controlled trials (RCTs), have investigated the role of mistletoe in cancer patients, showing benefits in terms of improved quality of life, performance index, symptom scales, and reduced adverse effects of chemotherapy [466]. A Cochrane review published in 2008, which included 21 studies, demonstrated the positive impact of mistletoe on various aspects of patient well-being [469]. Subsequent meta-analyses have further supported these findings, revealing that mistletoe extracts significantly improve global quality of life (SMD = 0.61, 95% CI 0.41–0.81, *p* < 0.00001) and may have a favorable effect on survival in cancer patients (HR = 0.81, 95% CI 0.69–0.95, *p* = 0.01) when used as an adjunct to conventional treatments [470]. A phase I trial of intravenous mistletoe extract in patients with advanced cancer showed a disease control rate of 23.8% and improved quality of life indicators. Mistletoe is commonly used by integrative oncologists to enhance quality of life, increase chemotherapy tolerability, and potentially contribute to better tumor control and survival [139].

#### 3.12.3. Dosing

Mistletoe has shown benefits in improving the quality of life in patients with various types of cancers, including breast, bladder, gynecological (cervical, corpus uteri, and ovarian), colorectal, gastric, pancreatic, glioma, head and neck, lung, melanoma, and osteosarcoma. However, as mistletoe is administered parenterally (subcutaneously or intravenously), it requires supervision by an integrative oncologist as part of a personalized treatment protocol [471].

### 3.13. Ashwagandhia

Ashwagandha (*Withania somnifera*, WS), historically employed in Mediterranean and Ayurvedic medicine, functions as both a functional food and medicinal plant with potential anticancer attributes [472]. Its active compounds, including withanolides and alkaloids, underpin its pharmacological effects [140].

#### 3.13.1. Mechanism

Preclinical investigations highlight Ashwagandha’s capacity to modulate mitochondrial function, facilitate apoptosis, and mitigate inflammation by targeting cytokines, nitric oxide, and reactive oxygen species [140,141,142]. It significantly contributes to apoptosis induction, suppresses cell proliferation and migration [140,141,142], and prompts cell cycle arrest and apoptosis in glioblastoma cells [473]. Notably, Ashwagandha’s impact extends to molecular pathways like p53, insulin/IGF, STAT3, and Notch [474,475,476]. Its anti-inflammatory potential can significantly alter the tumor microenvironment, curtailing angiogenesis and metastasis [477]. An intriguing study proposes that combining Ashwagandha extract with intermittent fasting could emerge as a promising approach for breast cancer treatment, effectively curbing cell proliferation, inducing apoptosis, and ameliorating cisplatin-related toxicity [142].

#### 3.13.2. Clinical Evidence

In the context of cancer, Ashwagandha has been primarily investigated through experimental models, with limited clinical data on its efficacy. Biswell et al. conducted an open-label prospective nonrandomized trial involving breast cancer patients, administering a combination of chemotherapy and Ashwagandha or chemotherapy alone. The study group receiving Withania somnifera root extract exhibited significantly lower fatigue levels and improved quality of life scores. Although the 24-month overall survival rates were higher in the study group (72%) compared to the control group (56%), the difference was not statistically significant [143]. Apart from its potential in cancer therapy, Ashwagandha is recognized as a safe and effective adaptogen, supported by randomized controlled trials showcasing its stress reduction, cognitive enhancement, mood improvement, and sleep quality benefits [307,308,309]. A meta-analysis of 12 trials demonstrated its significant reduction of anxiety (*p* = 0.005) and stress levels (*p* = 0.005) compared to placebo [144]. Although Ashwagandha’s impact on cancer outcomes remains unproven, its positive effects on stress, sleep, and quality of life suggest its potential as a recommended therapy for cancer patients.

#### 3.13.3. Dosing

Ashwagandha may be effective against cancers such as breast, colon, lung, prostate, glioblastoma multiforme, melanoma, and blood cancers [140,472]. Ashwagandha can be used to treat cancer alone or in combination with other chemotherapeutic agents [472].

### 3.14. Phosphodiesterase 5 Inhibitors

Selective phosphodiesterase 5 inhibitors, including sildenafil, tadalafil, and vardenafil, are widely used in the treatment of erectile dysfunction and pulmonary arterial hypertension [478]. These drugs may also be effective cancer treatments [479].

#### 3.14.1. Mechanism

PDE5 inhibitors, such as sildenafil and tadalafil, have shown promising anticancer effects in various types of cancers [432]. These inhibitors induce apoptosis and attenuate Wnt/β-catenin-mediated transcription in breast tumor cells, affect HSP90 expression to inhibit cancer cell proliferation, and reduce the development and progression of hepatocellular carcinoma induced by aflatoxin B1 [480]. Additionally, PDE5 inhibitors alter epithelial homeostasis, reduce polyp formation, and promote autophagy, leading to enhanced cell death when combined with cytotoxic agents [479,481]. They have also been shown to interact in a greater than additive fashion with NSAIDs, platinum-based chemotherapeutic agents, and curcumin, increasing their efficacy in controlling colorectal and lung tumors [482,483]. Moreover, PDE5 inhibitors can inhibit colonic tumorigenesis by blocking the recruitment of MDSCs, reducing Tregs and cancer stem cells, and inducing PKA signaling to eliminate cancer stem cells [479,484,485]. 

#### 3.14.2. Clinical Evidence

Several studies have demonstrated the potential anticancer benefits of PDE5 inhibitors. A large study involving 192,661 patients showed that PDE5 inhibitor use was associated with a reduced risk of developing colon cancer, and in men with benign colorectal neoplasms, it was associated with a lower risk of colorectal cancer [486]. Clinical trials in patients with head and neck squamous cell carcinoma revealed that tadalafil could enhance immune responsiveness and tumor-specific immunity by reducing MDSCs and regulatory T cells, and improving T-cell function [487,488]. In patients with colorectal cancer and prostate cancer, the post-diagnostic use of PDE5 inhibitors was associated with a decreased risk of cancer-specific mortality, metastasis, and biochemical recurrence [489,490].

#### 3.14.3. Dosing

Phosphodiesterase 5 inhibitors, such as sildenafil and tadalafil, may be beneficial for the treatment of prostate, breast, hepatocellular, colorectal, lung, and head and neck cancers, glioblastoma, and leukemias [479]. The recommended dosing includes sildenafil 20 mg daily or tadalafil 5 mg daily, but caution is advised in patients receiving nitrates or with a history of non-arteritic anterior ischemic optic neuropathy due to potentially serious cardiovascular side effects [149].

### 3.15. Itraconazole

Itraconazole, a well-established antifungal agent inhibiting lanosterol 14α-demethylase, has demonstrated potential as an anticancer agent through mechanisms unrelated to its antifungal effects.

#### 3.15.1. Mechanism

Its anticancer activity involves the reversal of P-glycoprotein-mediated chemoresistance, modulation of the Hedgehog, mTOR, and Wnt/β-catenin pathways, angiogenesis and lymphangiogenesis inhibition, and potential interference with cancer-stromal interactions [150]. Mechanistically, it impedes P-glycoprotein, disrupts abnormal Hedgehog and Wnt/β-catenin signaling, hinders angiogenesis, and triggers autophagocytosis [150,151,152,153,154,155,156,157,158,159,160]. Itraconazole further suppresses the PI3K/AKT/mTOR/S6K pathway, affecting cancer cell growth and proliferation [151,155,491], and inhibits HER2/Akt signaling by reducing HER2 phosphorylation [492]. Its induction of apoptosis is attributed to ROS pathway activation and death receptor pathway stimulation [155]. The drug curbs angiogenesis by obstructing VEGF/VEGFR2 interaction and endothelial cell cycle progression [158,491]. Itraconazole’s multifaceted modes of action suggest its potential as an innovative anticancer therapy beyond its antifungal properties [150,151,152,153,154,155,156,157,158,159,160,491].

#### 3.15.2. Clinical Evidence

Itraconazole demonstrates potential anticancer efficacy either as a single agent or in combination therapy based on preclinical and clinical data [151,152,153,154,156,157,159,160,493,494,495,496,497,498,499]. Notably, a phase II clinical study with lung cancer patients showed that itraconazole combined with conventional chemotherapy (pemetrexed) significantly improved progression-free and overall survival, which was attributed to its anti-angiogenic effects [151]. Retrospective studies supported the survival advantage of itraconazole treatment in refractory malignancies, including ovarian clear cell, triple-negative breast, pancreatic, and biliary tract cancer, compared to previous reports [493,497,498,500]. Clinical trials involving progressive pancreatic cancer and metastatic castration-resistant prostate cancer indicated positive outcomes with itraconazole-based combination treatments [156,497]. Moreover, itraconazole displayed concentration-dependent anticancer effects in non-small-cell lung cancer patients [499]. The drug’s potential adjuvant role was identified in various cancers, encompassing prostate, pancreatic, lung, breast, acute myeloid leukemia, basal cell carcinoma, medulloblastoma, hepatocellular carcinoma, esophageal, and gastric cancer [150,151,153,154,155,157,160,492,494,495,497].

#### 3.15.3. Dosing

Itraconazole in a dose of 400–600 mg/day is recommended. Itraconazole is a conventional antifungal drug that has received FDA approval and has an excellent safety record [151]. However, several studies have suggested that itraconazole has some contraindications, particularly when it comes to interactions with other cancer medications including rituximab or statins [501,502].

## 4. Potential Adjunctive Therapies

Adjunctive therapies demonstrate some potential for use in the treatment of cancer. These are summarized in Table 2 and the associated infographic Figure 2.

### 4.1. Tumor Treating Fields

Tumor treating fields (TTF) are non-invasive alternating electric fields administered via the Optune^®^ system, utilizing transdermally transmitted 100–400 kHz AC electric fields through orthogonal transducer arrays to disrupt mitosis [244,503]. This disrupts the mitotic spindle assembly checkpoint and leads to cell-cycle arrest, cell death, or senescence, while also promoting autophagy and immunological effects such as STING pathway activation and enhanced dendritic cell and macrophage activity [244]. Although extensively studied in glioblastoma multiforme (GBM), the use of TTF is being evaluated in NSCLC, pancreatic, and ovarian cancer [244]. In GBM, TTF in combination with maintenance temozolomide demonstrated significantly improved progression-free survival and overall survival [504]. The National Comprehensive Cancer Network (NCCN) recommends the use of TTF combined with temozolomide for both newly diagnosed and recurrent glioblastoma patients, suggesting it as an adjunctive treatment option [505,506]. Compliance is crucial as TTF’s therapeutic effects are limited to actively dividing cancer cells during its application [503].

### 4.2. Photodynamic Therapy

Photodynamic therapy (PDT) involves tissue destruction through visible light when combined with a photosensitizer and oxygen [507]. When exposed to light, sensitizer molecules transition to high-energy states, interacting with oxygen to produce reactive oxygen species that induce cell death through apoptosis, necrosis, and autophagy [508]. Historical use of light for therapeutic purposes dates back thousands of years, particularly combined with reactive chemicals to treat conditions like vitiligo, psoriasis, and skin cancer. Sunlight, encompassing ultraviolet-B (UVB) and near-infrared (NIR) radiation, offers significant health benefits including vitamin D synthesis and mitochondrial melatonin production [509,510]. However, modern lifestyles lead to deficient NIR exposure [510]. NIR-A radiation, with deep tissue penetration, demonstrated efficacy during the 1918 influenza pandemic, and recent studies link sun avoidance to higher all-cause mortality rates [511,512]. PDT, widely used by dermatologists for actinic keratoses and nonmelanoma skin cancers, holds potential for broader applications, including solid tumors, achieved through preferentially accumulated sensitizers activated by light [507,508]. Topical photosensitizers such as 5-aminolevulinic acid or methyl aminolevulinate are commonly employed for cutaneous indications, while visceral tumors require agents like porfimer sodium [507,513]. PDT’s efficacy in experimental cancer cell destruction is proven, yet clinical evidence supporting its benefits in non-cutaneous malignancies is limited [513,514,515,516]. PDT’s role in non-cutaneous cancer and photobiomodulation necessitates further assessment. To enhance mitochondrial function, regular midday sun exposure is recommended (at least three times a week), ideally through brisk walks [513].

### 4.3. Hyperbaric Oxygen

Hypoxia is a critical hallmark of solid tumors, associated with enhanced cell survival, angiogenesis, glycolytic metabolism, and metastasis [246]. Hyperbaric oxygen treatment (HBOT) has been employed for centuries to address hypoxia-related disorders, enhancing plasma oxygen levels and tissue delivery of oxygen [246]. HBOT induces hyperoxia and elevated reactive oxygen species (ROS), overwhelming cancer cell defenses and triggering cell death [517,518]. This process involves intricate signaling through protein kinases and receptors such as RAGE, CXCR2, TLR3, and TLR4 [519]. Despite limited direct impact on cancer growth, HBOT may synergize with other treatments; for instance, a ketogenic diet combined with HBOT exhibited significant anticancer effects [12]. Hypoxia contributes to chemoresistance, and HBOT as an adjuvant has demonstrated enhanced effects both in vitro and in vivo, although certain chemotherapeutic agents might interact negatively [246]. Radiotherapy combined with HBOT serves therapeutic and radiosensitizing purposes, particularly for head and neck tumors [246]. A recent Cochrane review cautioned that while HBOT might improve local tumor control and mortality for head and neck tumors, its benefits should be interpreted cautiously due to unusual fractionation schemes [520]. While HBOT holds promise as an anticancer intervention, particularly in combination with other modalities, clinical data supporting its efficacy remain limited [246].

## Figures and Tables

**Table 1:** A summary table of repurposed drugs including their mechanism, evidence for treatment efficacy, toxicity information and typical dosages. Italicized interventions are possible alternatives to the above interventions.

Intervention	Mechanism	Evidence	Toxicity	Dosage Per Day
**Metabolic and Lifestyle Interventions for Cancer Treatment**				
Glucose management and ketogenic diet	Restricting carbohydrates prevents their conversion to glucose, impacting the body’s metabolic and energy functions [38,39].	Prevent the high glucose spikes that fuel cancer [40].	Some possible complications for select populations.	Ketogenic diet (<50 g carbohydrates per day) in 8 h eating window [41].
Exercise	Multiple mechanisms [42].	Improved survival outcomes [43,44,45,46,47,48].	Possible overuse.	30 min per day [49].
Stress Reduction	Multiple mechanisms [50].	Stress is associated with higher cancer risk and poorer survival outcome [51,52,53].	No known fatalities.	Daily mindfulness.
Sleep	Multiple mechanisms [54].	Healthy sleep is essential for metabolic regulation [55].		7–8 h per night for adults [56].
**Tier One Repurposed Drugs—Strong Recommendation**				
Vitamin D	Inhibiting angiogenesis [57]. Stimulating adherence of cells [58]. Enhancing intercellular communication [59,60].	Statistically significant reductions in cancer mortality [61]. More significant reductions in cancer risk for those with normal BMI (<25) [62].	Serum 25-hydroxyvitamin D higher than 150 ng/mL are hallmark of vitamin D toxicity due to hypercalcemia [63].	20,000 to 50,000 IU daily [64,65].
Melatonin	Multiple mechanisms [66,67].	Low levels of melatonin increase breast cancer risk [68]. Increases cancer remission and survival rates [69].	Oral LD50 in mice: 1.25 g/kg [70].	Start at 1 mg and increase to 20–30 mg at night (extended/slow release) [71].
Green tea catechins	Inhibition of mitochondrial glutamate dehydrogenase by epigallocatechin gallate [72]. Suppression of cancer stem cells [73,74].	Green tea drinkers have lower risk of breast cancer [75]. Lowers risk of multiple cancers [76]. Lowers risk of non-Hodgkin’s Lymphoma [77].	EGCG: Mouse oral LD50 2.2 g/kg.	500–1000 mg daily of green tea extract [78].
Metformin	Blood glucose stabilization [79]. Inhibition of AMPK/mTOR pathway [80].	Lower incidence and higher survivability [81] of colorectal cancer [82]. Survival benefit for people with prostate cancer and concurrent diabetes [83]. Lower risk of cancer in people with type 2 diabetes [84].	Oral LD50 in rats: 1 g/kg [85].	1000 mg twice daily.
Curcumin	Inducing apoptosis selectively in cancer cells [86]. Multiple mechanisms [87].	Significant heterogeneity in trials depending on curcumin formulation [88].	Oral LD50 in rats: >5 g/kg [89].	400–600 mg daily [90] or as per manufacturer’s suggested dosing.
Mebendazole	Inhibits cancer-associated signaling pathways [91].	Case reports show improvement [92,93]. Case series with related drug fenbendazole show promise in treating genitourinary malignancies [94].	Oral LD50 in mice: >1280 mg/kg [95].	100–200 mg daily [96].
Omega 3	Modulation of cyclooxygenase activity, alteration of membrane and cell surface receptor function [97,98].	Protective against breast cancer in Asian patients [99]. Lower levels of Omega 3 relative to Omega 6 associated with higher cancer mortality [100].	N/A Possible heavy metal toxicity from extreme overuse [101].	2–4 g/day [102].
Berberine	Multiple mechanisms [103,104].	Can reduce risk of colorectal cancer [105]. Reduces tumor volume in animal studies [106].	Mouse oral LD50: 329 mg/kg [107].	A daily dose of 1000–1500 mg or 500–600 mg two or three times daily [108].
Atorvastatin	Multiple mechanisms [109,110].	Improvement [111,112,113,114,115,116,117,118].	Oral LD50 in mice: >5 g/kg [119].	40 mg 2x/day [120].
*Simvastatin*	*Multiple mechanisms [109,110].*	*Case series shows simvastatin may increase radiosensitivity of cancer cells [121].* *Statin use in US population associated with lower cancer mortality [114].*	*Oral mouse LD50: 3 g/kg [122].*	*20 mg 2x/day as an alternative to atorvastatin [123].*
Disulfiram	Multiple mechanisms [124,125].	Reduces tumor activity in breast cancer [126].	Oral rat LD50: 9 g/kg [127].	80 mg 3x daily or 500 mg once daily [128,129].
Cimetidine	Interferes with tumor cell adhesion, angiogenesis, and proliferation [130].	Improvement in gastric cancer survivability [131]. Improvement in survivability of surgical treatment of colorectal cancers [132].	Oral rat LD50: 5 g/kg [133].	400–800 mg twice daily [134].
Mistletoe	Protein synthesis interference, cell-cycle inhibition, and inducing apoptosis [135,136].	evidence of the efficacy of mistletoe extracts in gastric and female genital cancer [137].	Peritoneal rat LD50: 1–3 g/kg for stem aqueous extract [138].	Given subcutaneously by an integrative oncologist. Typical dose 600 mg 3x/week [139].
Ashwagandha	Modulates mitochondrial function, facilitates apoptosis, mitigates inflammations [140,141,142].	Non statistically significant increase in 24-month survival rates [143]. A meta-analysis of 12 trials demonstrated its significant reduction of anxiety (*p* = 0.005) and stress levels (*p* = 0.005) compared to placebo [144].	Mice oral LD50: 2 g/kg [145].	2 g daily [146].
Sildenafil	Enhances drug sensitivity [147].	N/A	Increase in adverse events above 200 mg [148].	20 mg daily [149].
Itraconazole	Inhibits P-glycoprotein, disrupts abnormal Hedgehog and Wnt/β-catenin signaling, hinders angiogenesis, and triggers autophagocytosis [150,151,152,153,154,155,156,157,158,159,160].	Phase II clinical study on itraconazole demonstrated significant improvement in progression-free and overall survival combined with pemetrexed [151].	Rat oral LD50: >320 mg/kg [161].	400–600 mg daily [162].
**Tier Two Repurposed Drugs—Potential Therapeutic Agents**				
Low dose naltrexone (LDN)	Interfering with cell signaling [163]. Immunomodulation [164]. Anti-inflammatory [165].	Improvement in tumors including non-small cell lung cancer (NSCLC) [166]. suppress human ovarian cancer [167].	Oral mouse LD50: 1 g/kg [168].	1–4.5 mg daily [169].
Doxycycline	Inhibiting anti-apoptotic and angiogenic proteins [170].	N/A	Oral rat LD50: 2 g/kg [171].	100 mg daily (for cycles of 2 weeks—use sparingly) [172].
Spironolactone	Effects the hallmarks of immune protection, invasion, and metastasis activation, and cell death resistance [173].	Spironolactone dramatically decreased the incidence of prostate cancer in clinical investigations [174,175,176].	Oral mouse LD50: >1 g/kg [177].	50–100 mg/day [175].
Resveratrol	Induction of apoptosis [178]. Inhibition of cancer stem cells [179].	In vivo evidence for anti-cancer effect, high heterogeneity in humans [180].	Predicted oral rat LD50, 48 h: 870 mg/kg/day [181].	500 mg, 2x daily [182].
Wheatgrass	Inhibition of metastasis and angiogenesis. Induction of apoptosis [183].	N/A	No observed toxicity at >2 g/kg oral dose in mice for 14 days [184].	9 g fermented wheat germ extract daily [185].
Captopril	Inhibits angiogenesis and blocks neovascularization. May play a role in the decrease of metastases (741).	In vivo and in vitro anticancer activity [186,187].	Rat oral LD50: 7 g/kg [188].	Unknown, but typical dose 12.5 mg [189].

**Table 2:** **An overview of potential anticancer agents with a weaker evidentiary basis (tier three), and ones that are recommended against (tier four)**.

Intervention	Mechanism	Evidence	Toxicity	Dosage Per Day
**Tier Three Repurposed Drugs-Equivocal Evidence**				
Aspirin	Multiple mechanisms [190].	Protective effect on esophageal cancer [191], and other cancers [192].	Oral mouse LD50: 250 mg/kg [193].	325 mg daily [194].
Diclofenac	Multiple mechanisms [195].	Improved disease free survival in breast cancer surgery [196]. Normalizes skin lesions when applied topically [197]. Case studies shows improvement [198,199,200,201,202].	Mouse oral LD50: 170 mg/kg [203].	Diclofenac 75–100 mg daily as alternative to aspirin [196].
Nigella Sativa (thymoquinone)	p53, NF-κB, PPARγ, STAT3, MAPK, and PI3K/AKT signaling pathways [204].	Improved treatment outcome in acute lymphoblastic leukemia in children [205].	Oral mouse LD50: 29 mL/kg [206].	400–500 mg encapsulated oil twice daily, avoid during pregnancy [207].
Reishi	Immunomodulation [208,209].	50% increased effectiveness of cancer chemotherapy [210].	Oral mouse LD50: >10 g/kg [211].	6–12 g of Reishi extract per day [212].
Ivermectin	Regulates multiple signaling pathways [213,214].	Case series showed improvement in patient symptoms when combined with dichloroacetate [215].	Mouse oral LD50: >27 mg/kg [216].	12–60 mg 2x/week [215].
Dipyridamole	Increases tumor chemosensitivity [217].	Increases efficacy of other anti-cancer agents [218,219,220].	Rat oral LD50: 8 g/kg [221].	100 mg twice daily [222].
Intravenous Vitamin C	Targeted killing (through intracellular generation of H_2_O_2_) of cancer cells [223].	Improved life quality in cancer patients [224], lowered inflammation [225].	Rat intravenous LD50 >4 g/kg [226].	50–75 g IV as per protocol [227,228,229,230].
Dichloroacetate	Inhibits dehydrogenase kinase to inhibit metabolic reprogramming by cancer cells [231].	Greater treatment response, but no impact on survival [232].	Rat oral LD50 5 g/kg [233].	500 mg two/three times daily [234].
Cannabinoids	Induction of cancer cell death by apoptosis and inhibition of cancer cell proliferation [235].	Useful in treating refractory chemotherapy-induced nausea and vomiting. Case studies show possible benefit [236].	THC Mouse oral LD50: 500 mg/kg [237] CBD Mouse oral LD50: >100 mg/kg [238].	Daily doses range from 10 to 800 mg CBD and from 5 to 8 mg THC [239].
Fenofibrate	Stimulation of peroxisome proliferator activated receptor α (PPARα) [240].	No clinical data.	Mouse oral LD50: 1.6 g/kg [241].	N/A
Pao Pereira	Inhibition of NF kappa B Signaling [242].	Effectiveness in prostate cancer [243].	Limited information.	N/A
**Potential Adjunctive Therapies**				
Tumor Treating Fields	Multiple mechanisms, induction of apoptosis and autophagy [244].	N/A	N/A	
Photodynamic therapy	Direct cellular damage, vascular shutdown and activation of immune response against tumor cells [245].	N/A	N/A	
Hyperbaric Oxygen	Elevates levels of reactive oxygen species to signal cell death in cancer cells [246].	N/A	Possible adverse reactions [247].	
**Tier Four Repurposed Drugs—Recommend Against**				
Shark cartilage	Inhibition of angiogenesis. Sphyrnastatin 1 and 2 have anti-angiogenic activity and inhibit neovascularization [248].	N/A	Gastric adverse events [249], potential neurotoxicity [250].	
Laetrile (amygdalin)	Multiple mechanisms [251].	Produced few clinical side effects [252].	Rat oral LD50: 0.9 g [253].	

## Supplementary Materials

The following supporting information can be downloaded at: https://www.mdpi.com/article/10.3390/nu15194245/s1, Table S1: Cost of repurposed drugs for cancer treatment.
